# Immunopeptidomics toolkit library (IPTK): a python-based modular toolbox for analyzing immunopeptidomics data

**DOI:** 10.1186/s12859-021-04315-0

**Published:** 2021-08-17

**Authors:** Hesham ElAbd, Frauke Degenhardt, Tomas Koudelka, Ann-Kristin Kamps, Andreas Tholey, Petra Bacher, Tobias L. Lenz, Andre Franke, Mareike Wendorff

**Affiliations:** 1 Institute of Clinical Molecular Biology, Christian-Albrechts-University of Kiel, Kiel, Germany; 2 Proteomics and Bioanalytics, Institute for Experimental Medicine, Christian-Albrechts-University of Kiel, Kiel, Germany; 3 Institute of Immunology, Christian-Albrechts-University of Kiel, Kiel, Germany; 4 Research Unit for Evolutionary Immunogenomics, Department of Biology, University of Hamburg, Hamburg, Germany

**Keywords:** HLA, Immunopeptidomics, Antigen processing and presentation, Computational immunology, Interactive data analysis

## Abstract

**Background:**

The human leukocyte antigen (HLA) proteins play a fundamental role in the adaptive immune system as they present peptides to T cells. Mass-spectrometry-based immunopeptidomics is a promising and powerful tool for characterizing the immunopeptidomic landscape of HLA proteins, that is the peptides presented on HLA proteins. Despite the growing interest in the technology, and the recent rise of immunopeptidomics-specific identification pipelines, there is still a gap in data-analysis and software tools that are specialized in analyzing and visualizing immunopeptidomics data.

**Results:**

We present the IPTK library which is an open-source Python-based library for analyzing, visualizing, comparing, and integrating different omics layers with the identified peptides for an in-depth characterization of the immunopeptidome. Using different datasets, we illustrate the ability of the library to enrich the result of the identified peptidomes. Also, we demonstrate the utility of the library in developing other software and tools by developing an easy-to-use dashboard that can be used for the interactive analysis of the results.

**Conclusion:**

IPTK provides a modular and extendable framework for analyzing and integrating immunopeptidomes with different omics layers. The library is deployed into *PyPI* at https://pypi.org/project/IPTKL/ and into *Bioconda* at https://anaconda.org/bioconda/iptkl, while the source code of the library and the dashboard, along with the online tutorials are available at https://github.com/ikmb/iptoolkit.

**Supplementary Information:**

The online version contains supplementary material available at 10.1186/s12859-021-04315-0.

## Background

The human leukocyte antigen (HLA) complex, located on chromosome 6p21, is a hotspot for immune-system related genes [1]. The HLA loci contain, among others, the loci that encode for the classical HLA class I proteins, HLA*-A*, HLA*-B* and HLA*-C* and the classical HLA class II proteins, HLA*-DR*, HLA*-DP* and HLA*-DQ* [2]. HLA-I proteins present mainly peptides derived from the proteasome-digested proteins to CD8^+^ T-cells while HLA-II proteins present lysosome-digested proteins to CD4^+^ T-cells. From a genetic perspective, both HLA class I and class II are highly polymorphic with the majority of the allelic variation being located within the region encoding for the peptide-binding protein domain [3]. Different HLA alleles have been associated not only with a wide spectrum of autoimmune and inflammatory diseases, for example, inflammatory bowel disease [4, 5], multiple sclerosis [6] and systemic lupus erythematosus (SLE) [7], but have also been implicated in pharmacogenomics and precision medicine. It has been recently shown by Sazonovs et al. [8] that carriers of HLA-DQA1*05 alleles are more likely to develop anti-drug antibodies towards Infliximab and Adalimumab.

Hence, characterizing and identifying peptides presented by HLA proteins is of paramount importance. For example, it can be utilized in rational vaccine design and development [9], neoantigen identification and tumor immunotherapy [10, 11], and to provide a mechanistic understanding of HLA-disease association [2, 12]. To this end, different *in silico* tools and experimental methods have been developed for characterizing and identifying peptides presented by HLA proteins. However, within the last decade, mass-spectrometry (MS)-based methods have become the default method for characterizing the peptides presented by HLA proteins *in vivo*, referred to as the immunopeptidome [13–15].

The workflow of an immunopeptidomics pipeline starts with the immunoprecipitation of the HLA-peptide complex using HLA-specific antibodies, for example, L243 for HLA-DR [16–18] and W632 for HLA-I [18, 19]. Next, the bound peptides are disassociated from their pulled HLA proteins by acid denaturation, followed by the purification of the peptides using chromatographic techniques. Finally, the purified peptides are analyzed using a wide variety of liquid chromatography tandem mass spectrometry (LC–MS/MS) protocols and techniques [18].

Computationally, the first step in the analysis is the processing of the generated spectra followed by the derivation of peptide sequences, using preexisting proteomics tools, for example, *MaxQuant* [20], *Mascot* [21], *x!!Tandem* [22], and *OMSSA* [23]. However, given the differences between standard proteomics and immunopeptidomics, e.g., the lack of trypsin digestion in the latter, a wide range of immunopeptidome-tailored identification pipelines and tools have been developed. For example, *MHCQuant* [24] and *NeoFlow* [25] which are tailored for neo-epitopes discovery, and *NewAnce* [26], which is tailored for handling non-canonical tumor immunopeptidomes. Nevertheless, to the best of our knowledge, there are no specific tools for the downstream analysis of immunopeptidomes identification pipelines.

Hence, to facilitate the analysis of the fast-growing number of immunopeptidomics datasets, we here present the **i**mmuno**p**eptidomics **t**ool **k**it library, IPTK. The library is implemented in Python and utilizes its rich collection of data-science tools and libraries to provide a large number of modular units that can be used for analyzing, comparing, and visualizing the results of identification pipelines. It can also be used for integrating different omics layers, for example, the transcriptome, with the list of identified peptides to deliver a richer biological meaning of the results. The modular units of the library can be combined variably to fit the unique requirement of each experiment, or they can act as building blocks for developing other analysis tools and pipelines. The library is extensively documented with online tutorials that cover different use cases.

## Implementation of IPTK

### IPTK design and structural components

**Fig. 1:**
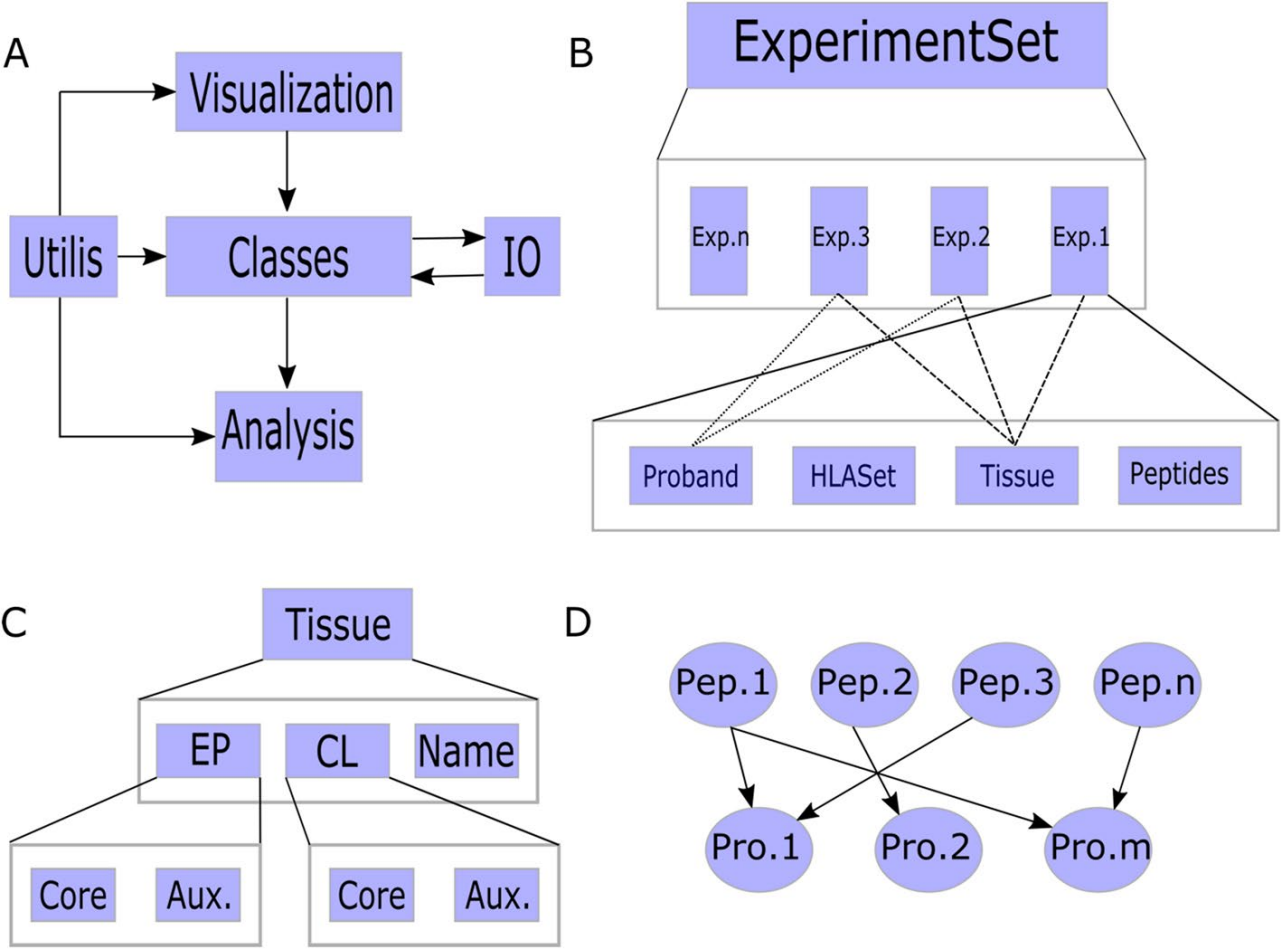
An overview of the IPTK library structure and design. **A** A high-level view of its different modules and how they interact with each other. **B** An overview of the Classes module and its hierarchy. *ExperimentSet* represents the highest level of abstraction in the module, each *ExperimentSet* is a collection of experiments, donated by *Exp.1* to *Exp. n*, each of which composites mainly of; *Proband*, *HLASet*, *Tissue* and *Peptides*. **C** The API of the *Tissue* class where EP is the expression profile, CL is cellular location and Aux. is the auxiliary gene expression and protein localization, respectively. **D** The IPTK abstraction of the mapping between peptides and proteins as acyclic unidirectional graph

The immunopeptidomics toolkit, IPTK, library is a python-based library that provides a framework for analyzing immunopeptidomics data and integrating different omics layers, for example, transcriptomics, sub-cellular compartment, and 3D structure data with the list of identified peptides for a rich downstream analysis of the results. IPTK depends on *Matplotlib* [27] and *Plotly* [28] for visualization, *NumPy* [29] for computation, *Pandas* [30] for handling and storing data and *Biopython* [31] for loading and parsing biological data. Structurally, IPTK is composed of five main modules as shown in Fig. 1A, the *Input–Output* (*IO)* module, the *Classes* module, the *Analysis* module, the *Visualization* module and the *Utilis* module.

The *IO* module provides functions to parse and read a wide variety of data formats used by the proteomics community for peptide identification, for example, *pepXML, mzIdentML* and *idXML* through the utilization of the *Pytomics* library [32]. The *Classes* module is the core engine of the library. It encapsulates and provides high-level abstractions for processing, integrating, and analyzing the data. It can be subdivided into different submodules that abstract different parts of the immunopeptidomics experiments. The *Experiment* class provides an abstraction for different mass-spectrometry runs, same experiment but different database search engines, for example, *Comet* [33] or *MS-GF* + [34], or completely different experiments. It also acts as the anchor point for linking different components of an experiment, for example, it links HLA types with gene expression, peptide identification, cellular component, and sample metadata. The *ExperimentSet*, provides an abstraction for a collection of experiments and provides different analysis tools: i.e. methods to compare chosen entities, e.g. comparing protein coverages among different experiments, methods to combine chosen entities, e.g. combine peptides and proteins of different experiments; methods to filter chosen entities: e.g. extract only peptides and proteins identified in all experiments, and methods to group chosen entities, e.g. group experiments obtained from the same tissue or the same sample together (Fig. 1B). Similarly, the classes *MzMLExperiment*, and *MzMLExperimentSet* can be used to abstract the parsing and analysis of the raw *MzML* files by acting as a wrapper for the *PyOpenMS* library [35]. Thus, enabling the integration of spectral information with the identified peptides and other omics layers.

The *Tissue* class provides abstraction for the source of the tissue or cell-culture. It abstracts a tissue into three major components, the first is the name of the tissue, the second is the *Expression Profile*, EP, which summarizes information about the gene expression in the provided tissue and the third is the *Cellular Location*, CL, which summarizes information about the subcellular compartment of the tissues’ proteins. Both EP and CL distinguish between core proteins which are the major components of the tissue and auxiliary proteins which might be added to the tissue as media proteins or non-host related proteins (Fig. 1C). The classes *Peptide* and *Protein* provide abstraction for the identified peptides and inferred proteins, respectively, along with a mapping between them (Fig. 1D). Finally, the *Classes* module additionally contains other classes that are used throughout the library, for example, the *Database s*tores and defines different data containers while the *Features* class provides an easy-to-use and easy-to-program interface for extracting and manipulating all known information about the proteins in UniProt [36]. Finally, the *GOEngine* acts as a wrapper for *GOATOOLS* [37] enabling gene ontology enrichment analysis (GOEA) to be seamlessly conducted on the identified proteins.

The *Analysis* module contains all the functions used by the *Classes* and *Visualization* modules, while the *Utility* module contains utility and helper functions used throughout the library. Finally, the *Visualization* module contains functions that can be used for visualizing the results generated by the library. The visualization functions are implemented using *Matplotlib* [27], *Seaborn* [38] and *Plotly* [28] to address different use cases. For example, *Plotly-based* functions can be seamlessly integrated with *Dash* framework to build powerful interactive dashboards. While, *Seaborn* and *Matplotlib* can be easily integrated with *Juypter Notebook* [39]. Thus, the library can easily blend with the two most widely used data analysis and visualization frameworks in Python.

IPTK also, introduces some novel methods to visualize the results computed by the analysis functions. For example, paired coverage representation which compare the coverage of the same protein in two different conditions or its generalization the n-coverage representation, which visualizes the coverage among arbitrary number of conditions. A second example is the coverage-and-annotation plot which combines protein coverage with pre-existing knowledge available on UniProt [36].

Finally, to link peptide presentation with the protein 3D structure, the library uses imposed representation where a cartoon representation of the protein is used to capture the 3D structure and the coverage array (*Immunopeptidomic-coverage as a distance metric)* is used to construct a color gradient to color each amino acid in the protein according to its coverage. The imposed representation depends on *NGLViewer* [40] and *Jupyter Notebook* [39] to provide an interactive analysis of the generated representation on web-browsers.

### Immunopeptidomic-coverage as a distance metric

Immunopeptidomic-coverage is a concept similar to the depth used with DNA and RNA sequencing. IPTK defines the coverage as the number of unique immunopeptides that cover a specific position, i.e., a single amino acid position, in the parent protein. Internally, IPTK represents the coverage of each parent protein as an array that has the same length as the parent protein with each element of the array representing the coverage at the corresponding position in the parent protein. Hence, the difference in coverage for the same protein among different conditions can be computed as the sum of the absolute difference between the corresponding coverage arrays in these conditions. Thus, proteins with similar coverage will have lower scores while proteins with dissimilar coverage will have a large score. Finally, the library generalizes this concept to compute the distance among experiments, by averaging the scores over all proteins.

### Integrating immunopeptidomics and transcriptomic data

As stated above, the *Tissue* class is used as an abstraction for the source tissue, i.e., the tissue from which peptides have been eluted. A core component of the tissue class is the gene expression profile of the abstracted tissue which is a table that contains the expression value for each gene in the specified tissue. IPTK allows users to provide their own gene expression table, otherwise it uses a default table obtained from the Human Protein Atlas [41].

Once the transcriptomics layer has been linked with the immunopeptidomics layer, a wide range of functions can be used to extract biological insights about the mapping between the two layers. For example, comparing the gene expression of the proteins that were inferred from the immunopeptidome and the non-presented proteins which can provide more insights about the impact of gene expression on the composition of the immunopeptidome in the tissue and condition under-investigation. Alternatively, this information can be exported and used to construct predictive HLA-peptide binding models that combine both layers to extrapolate this knowledge to new HLA alleles, or un-studied tissues [42].

### Integrating immunopeptidomics and sub-cellular compartment data

On the contrary to proteomics, where all the proteins in a sample are digested and analyzed, immunopeptidomics solely focuses on the set of pre-selected and pre-digested peptides by the HLA processing machinery. Hence, factors governing the selection of these proteins are of paramount importance to understand the immunopeptidome formation. One of these factors might be the sub-cellular compartment which can control the accessibility of the HLA-processing machinery to the protein. This is especially prominent for the case of HLA-II where availability at the lysosomal compartment is a prerequisite. Hence, IPTK provides support to link the protein sub-cellular compartment with the immunopeptidome and other omics layers. This is achieved through the abstraction provided by the *Tissue* class, which operates in the same manner as the transcriptome layer, defined above. Once this layer has been linked, the number of peptides and inferred proteins observed from each compartment can be calculated and compared among different experiments. Data on sub-cellular compartments are either derived from the Human Protein Atlas [41] or can be provided by the user. Thus, the *Tissue* class provides methods for obtaining the subcellular compartment of each protein, while the class *GOEngine (IPTK design and structural components),* provides methods to agglomerate cellular component information and provides an overview about the enrichment of each component in the immunopeptidome.

### Integrating immunopeptidomics and protein structure

As discussed above, usually proteolytic digestion is an essential step in bottom-up proteomics. This step is omitted in immunopeptidomics. Indeed, the factors governing the cleavage of proteins are of paramount importance for understanding antigen processing and presentation. Different factors might contribute to processing and presentation, for example, the cell type and the processing machinery as explained above but also protein specific factors, for example, the 3D structure of the protein and its post-translational modification (PTM).

To enable the integration of the 3D structure with the immunopeptidome, IPTK has a built-in support to download and extract 3D structure information available on Protein Data Bank (PDB) [43]. This is achieved by first querying the mapping services of UniProt to map UniProt IDs to the PDB IDs. In case of multiple mapping, i.e., more than one PDB ID per UniProt ID, the first PDB ID is selected. Alternatively, the user can choose which ID to use or to skip the mapping step and provide the PDB identifier directly. Once the IDs have been obtained, *Biopython* is used to download and parse the 3D structure data. Finally, IPTK toolbox is used to analyze the results and integrate it with other omics layers defined above.

### Integrating immunopeptidomics and taxonomic data

As stated above, immunopeptidomics provides a powerful technology to capture the presented peptidome *in vivo*, which makes it an ideal technology to study host–pathogen interactions. This implies that in some experimental settings, annotating the immunopeptidome with an organism’s taxonomic information might provide insights about the pathogen or, generally, the non-host components of the immunopeptidome. To this end, IPTK provides a built-in support to annotate each inferred protein with its origin. This can be done using the *OrganismDB* class, which acts as a map to link each UniProt ID with an organism of origin. The constructor of the class can either be fed with a table containing the mapping or with the path to a FASTA file containing the sequences in a UniProt FASTA format, it then parses the file and construct a mapping table that can be used to annotate the inferred proteins. Once the proteins have been annotated, the library has a large collection of functions that can be used to subset, group, remove and count peptides and inferred proteins based on taxonomic information.

## Results

### IPTK workflow

**Fig. 2:**
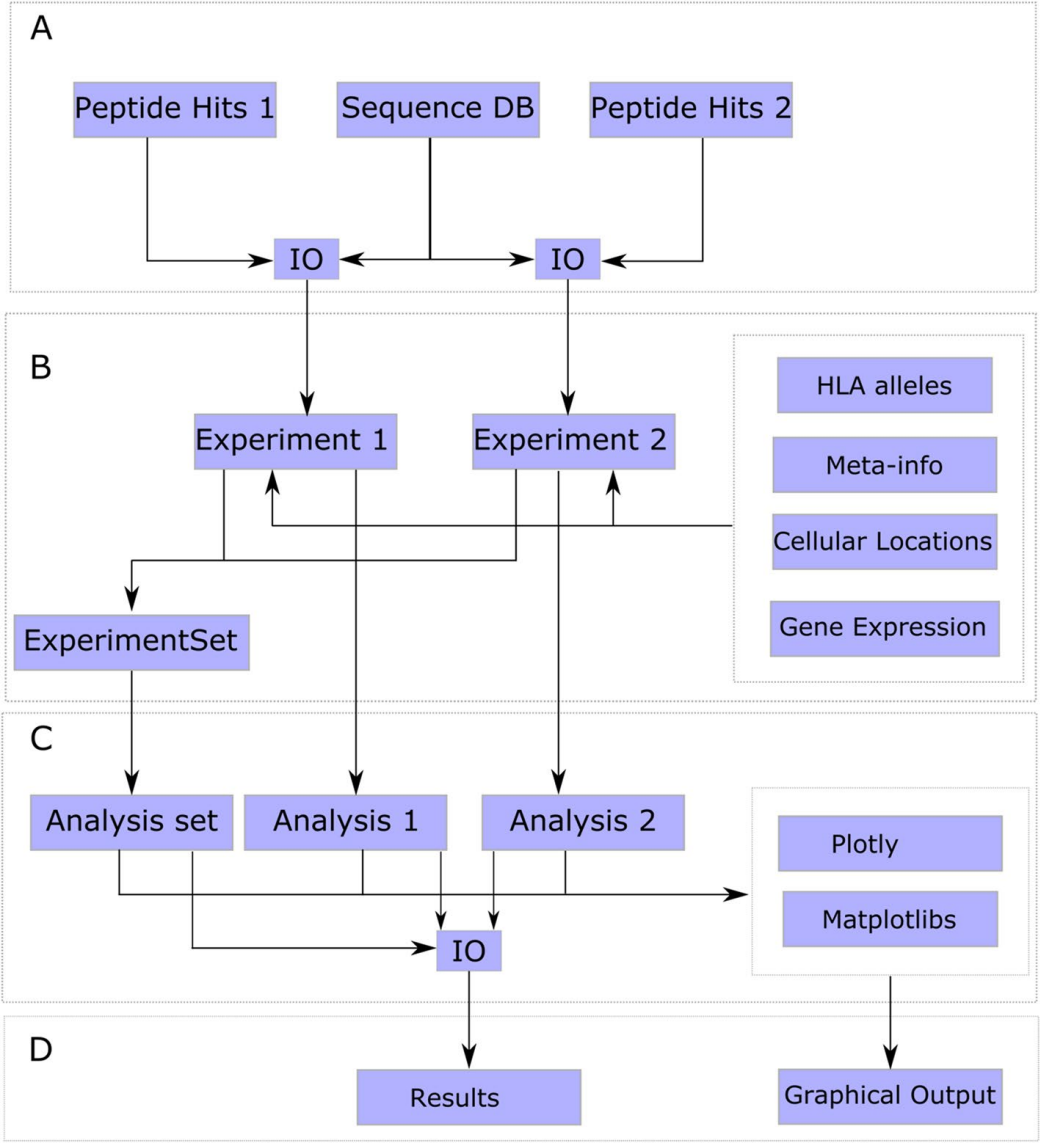
A typical workflow using the IPTK library*.* **A** The execution starts by providing a file containing the list of peptide identification hits, for example, in a *pepXML* or an *idXML* format, along with the sequences database used during the database search, these inputs are then processed and parsed by the *IO* module to generate a uniform internal representation of the input referred to as the identification table. **B** The generated identification table is combined with other information about the samples, for example, meta-information about the donors where the tissue(s) has/have been eluted to construct the *Experiment* object, and different *Experiment* objects can be combined to generate an *ExperimentSet* object. **C** The abstract objects constructed in (**B**) are then analyzed either using the built-in methods or through the functions defined in the analysis modules, each experiment can be analyzed individually, or they can be combined and compared through the *ExperimentSet* API as discussed above. **D** The results of the analysis executed on (**C**) can be exported graphically using either *Plotly* or *Matplotlib* libraries or can be written to a file using the *IO* module for further downstream analysis and integration

Figure 2 shows a typical analysis workflow using IPTK starting by parsing a list of peptide hits identified using the database search engine along with the sequences database. Followed by the construction of an *Experiment* and/or *ExperimentSet* object through the integration of the peptide hits with the tissue’s gene expression and/or cellular location, HLA alleles, meta information, et cetera. Once these objects have been constructed, all the analysis and visualization functions defined above can be applied.

### Use case 1: analyzing HLA-ligand atlas database

As a first case study, we started by analyzing data from the HLA-ligand atlas [44] database using the library. Given that different tissues have different processing capabilities, for example, by expressing different sets of digestive enzymes, we started first by looking at the sequences located upstream and downstream of the identified peptides in their inferred parent proteins. After the n-mers were extracted from the proteins, IPTKs interface to MEME software [45] was deployed to compute the motifs of the adjoined regions shown in Additional file 1: Fig. S1 and Fig. S2.

The observed difference in the motifs among tissues can be a consequence of different proteins being expressed or available, for example, present in the extra-cellular matrix, of different tissues. A second contributing factor might be the differential expression of digestive and processing enzymes. Interestingly, comparing the motif of the same tissue among different individuals (Additional file 1: Fig. S2) revealed considerable differences. This might be the result of HLA-variability, where different alleles bind to different subsets of the available peptide pool and hence different proteins or different parts of the protein are presented, leading to differences in the computed motif among individuals.

Previously, Chen et al. [42] have identified gene expression as a major contributing factor in shaping HLA-II immunopeptidome. To this end, we used IPTK to integrate the immunopeptidomes of different tissues available on the HLA-ligand atlas [44] with the transcriptome of these tissues using the Human protein Atlas [41] to analyze the impact of gene expression on shaping HLA-II peptidomes. As shown in Additional file 1: Fig. S3, there was a significant difference in the gene expression of the presented proteins and the non-presented proteins, confirming the previous finding of Chen and colleagues.

Next, we used IPTK library to compare the HLA-II immunopeptidomes among different tissues. Five different methods implemented in the library were used: (1) pairwise peptide-overlap (Additional file 1: Fig. S4), (2) peptide-level Jaccard index (Additional file 1: Fig. S5), (3) pairwise protein-overlap (Additional file 1: Fig. S6), (4) protein-level Jaccard index (Additional file 1: Fig. S7), and (5) pairwise immunopeptidomics coverage (*Immunopeptidomics-coverage as a distance metric*) (Additional file 1: Fig. S8). In the pairwise peptide overlap the number of peptides with exact match between each pair of tissues is used as a similarity metric, while in the pairwise protein-overlap, protein level overlap is used as the similarity metric. As the pair-wise based methods might be biased by the number of peptides or proteins identified in each experiment, IPTK supports Jaccard-based normalization to account for differences in the size of the immunopeptidome of different tissues. In IPTK, Jaccard-index is computed as the number of peptides or proteins identified in a pair of experiments, i.e. detected in both experiments, divided by the total number of unique peptides or proteins identified in the two experiments. As discussed above and shown here, these differences among tissues reflect the complexity of HLA-II processing machinery, which is sensitive to a wide range of factors, for example, protein expression level, protein trafficking to the endo-lysosomal compartment, the differential expression of processing enzymes and HLA-allelic variability.

**Fig. 3:**
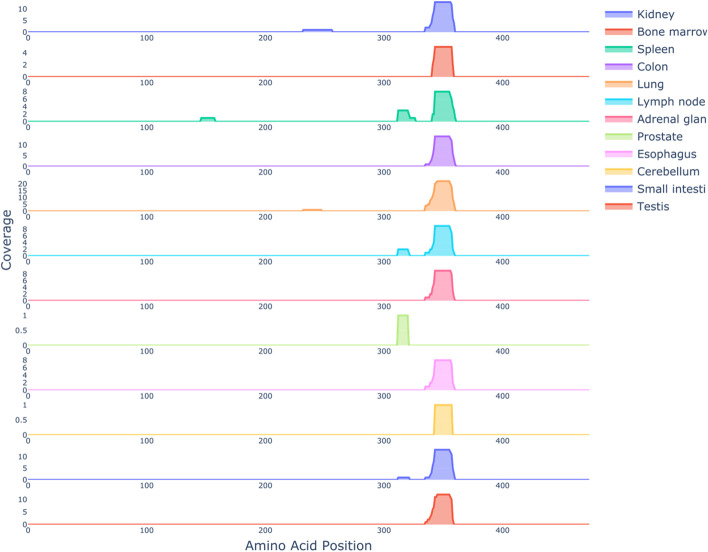
An n-coverage representation figure for the extra cellular protein Vitamin D-binding protein, UniProt ID P02774, across 12 different tissues for the donor AUT01-DN08. The immunopeptidome data were obtained from HLA-Ligand Atlas release 2020.6

Given the considerable differences in the peptidome of different tissues we were interested in quantifying the presentation of the same protein among different tissues. To this end, we used the n-coverage representation function (*IPTK design and structural components*) to plot the coverage array of the extra cellular protein Vitamin D-binding protein across 12 different tissues (Fig. 3). As shown in the figure, the presented part of the protein is ubiquitously presented among all tissues while other regions show a more tissue specific pattern. On one hand, this might be a reflection of the underlining processing machinery where some digestion enzymes are ubiquitously expressed while others show a more restrictive and tissue specific expression. On the other hand, this might reflect the homology and redundancy, where in some tissues a homologues protein is presented and due to homology or a shared protein-family with the protein under investigation, different parts of the protein is assumed to be presented.

### Use case 2: characterizing the impact of initial cell count on the identified immunopeptidome

**Fig. 4:**
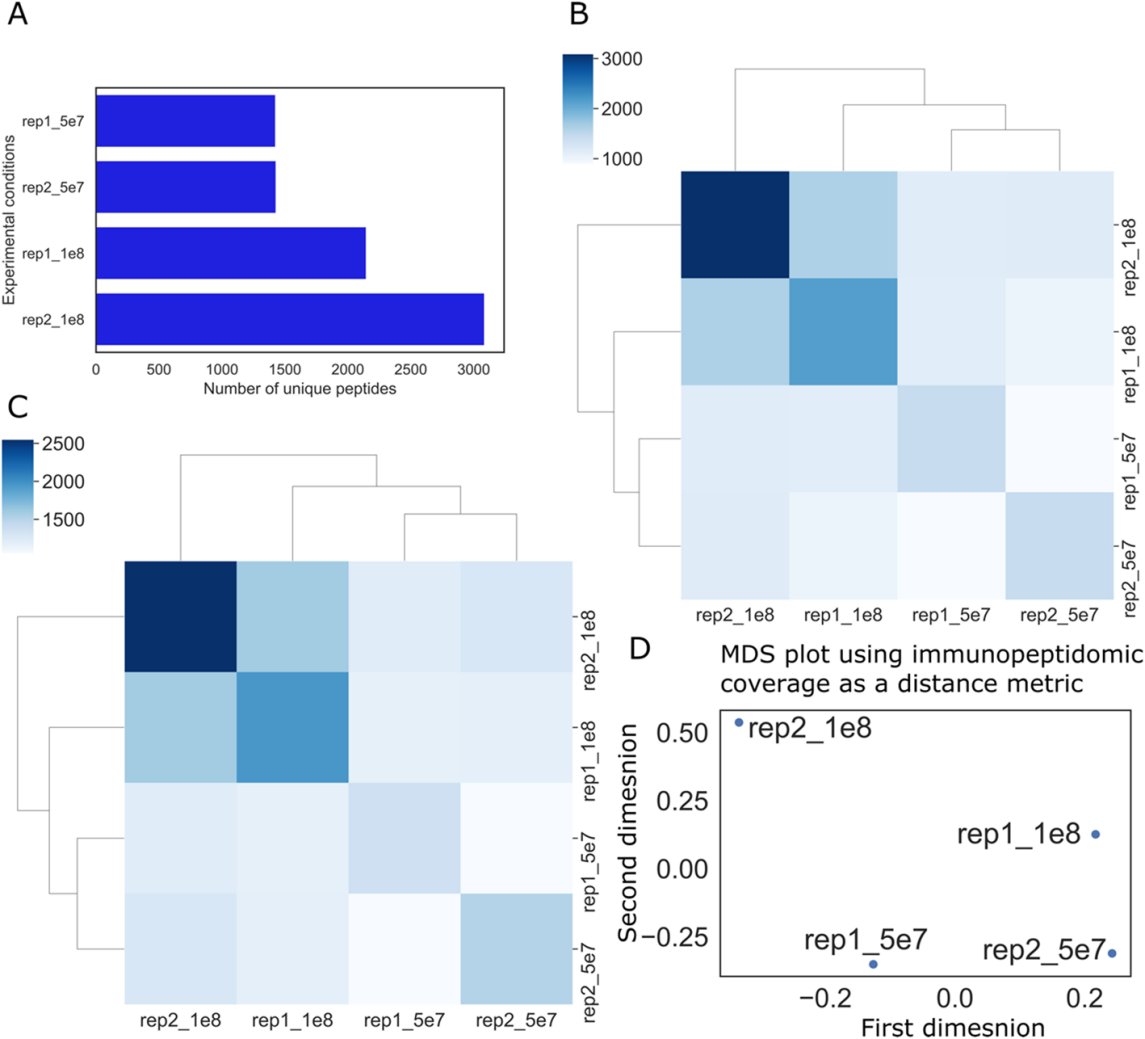
Analysis of HLA-DR immunopeptidomes using two replicates and two different staring cell counts. *5e7_R1* is the first replicate with 5 × 10^7^ cells, while *5e7_R2* is the second replicate with the same initial cell counts. *1e8_R1* is the first replicate with 1 × 10^8^ cells while *1e8_R2* is the second replicate using the same cell count. **A** is the number of unique peptides observed in each immunopeptidome. **B** is a Custer map based on peptide overlap between each pair of experiments. **C** is a cluster map based on protein overlap between each pair of experiments. **D** is a multi-dimensional scaling (MDS) plot using immunopeptidomics coverage as a distance metric

As a second case study, we used IPTK to study the impact of the initial cell count on the HLA-DR immunopeptidome. To this end, we captured the HLA-DR immunopeptidome of total peripheral blood mononuclear cells (PBMCs) starting from two initial cell counts, 5 × 10^7^ and 1 × 10^8^ cells (*Data Generation).* First, we started by analyzing, the number of peptides identified for each run (Fig. 4A). Second, we looked at the overlap among the four samples using pairwise peptide-overlap (Fig. 4B), pairwise protein-overlap (Fig. 4C) and pairwise immunopeptidomics coverage (Fig. 4D). As shown in Fig. 4A, increasing the initial number of cells is associated with increasing the number of peptides identified. Interestingly, the variation in the absolute number of identified peptides between replicates was higher at the higher cell number, i.e., 1 × 10^8^ cells. This might be the result of antibody saturation; however, more replicates are needed to test this hypothesis.

**Fig. 5:**
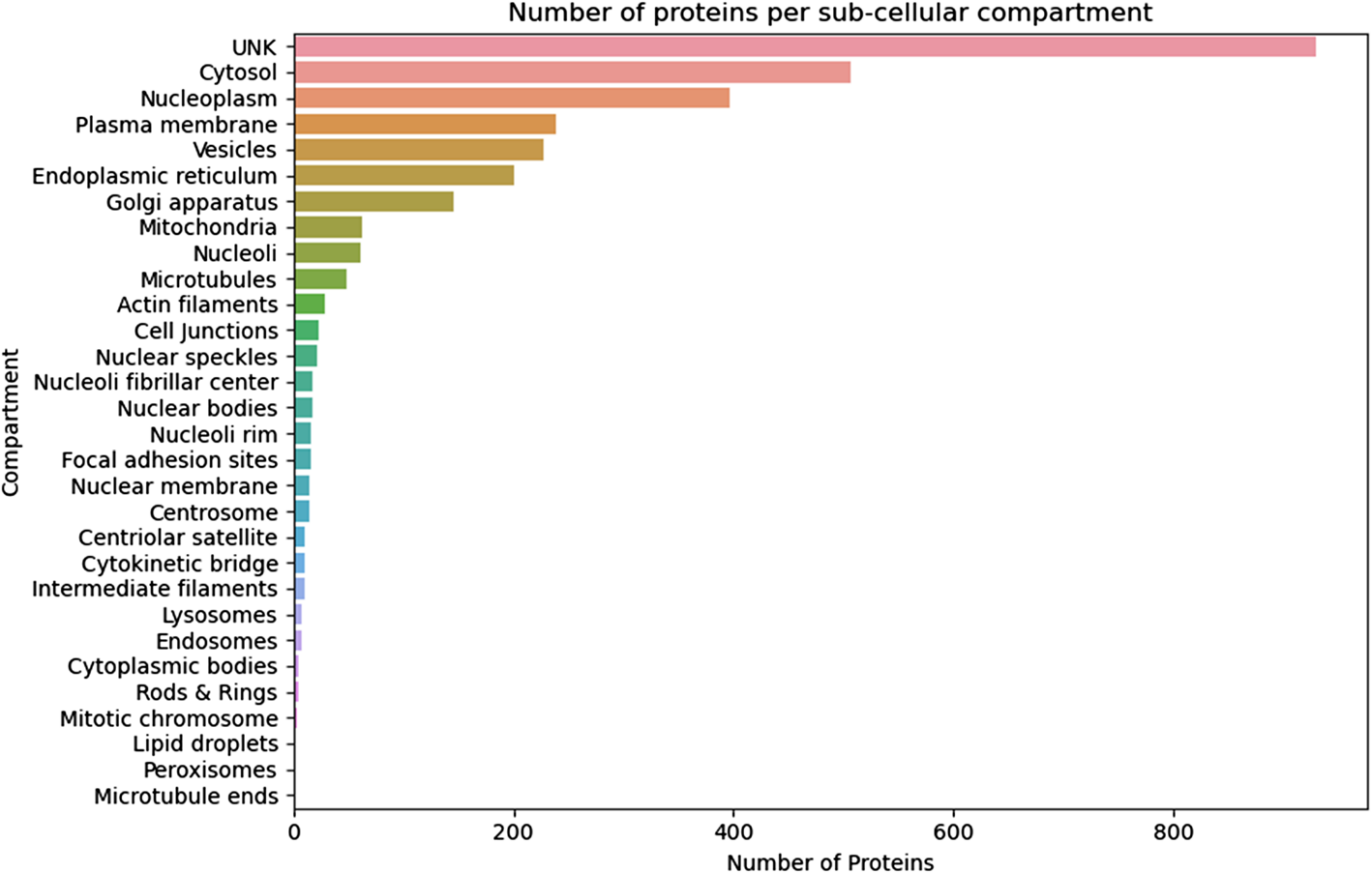
The distribution of proteins inferred from the HLA-DR immunopeptidome of total PBMC among different sub-cellular compartments. Data was generated from 1 × 10^8^ cells and using the protocol described at (Data Generation)

**Fig. 6:**
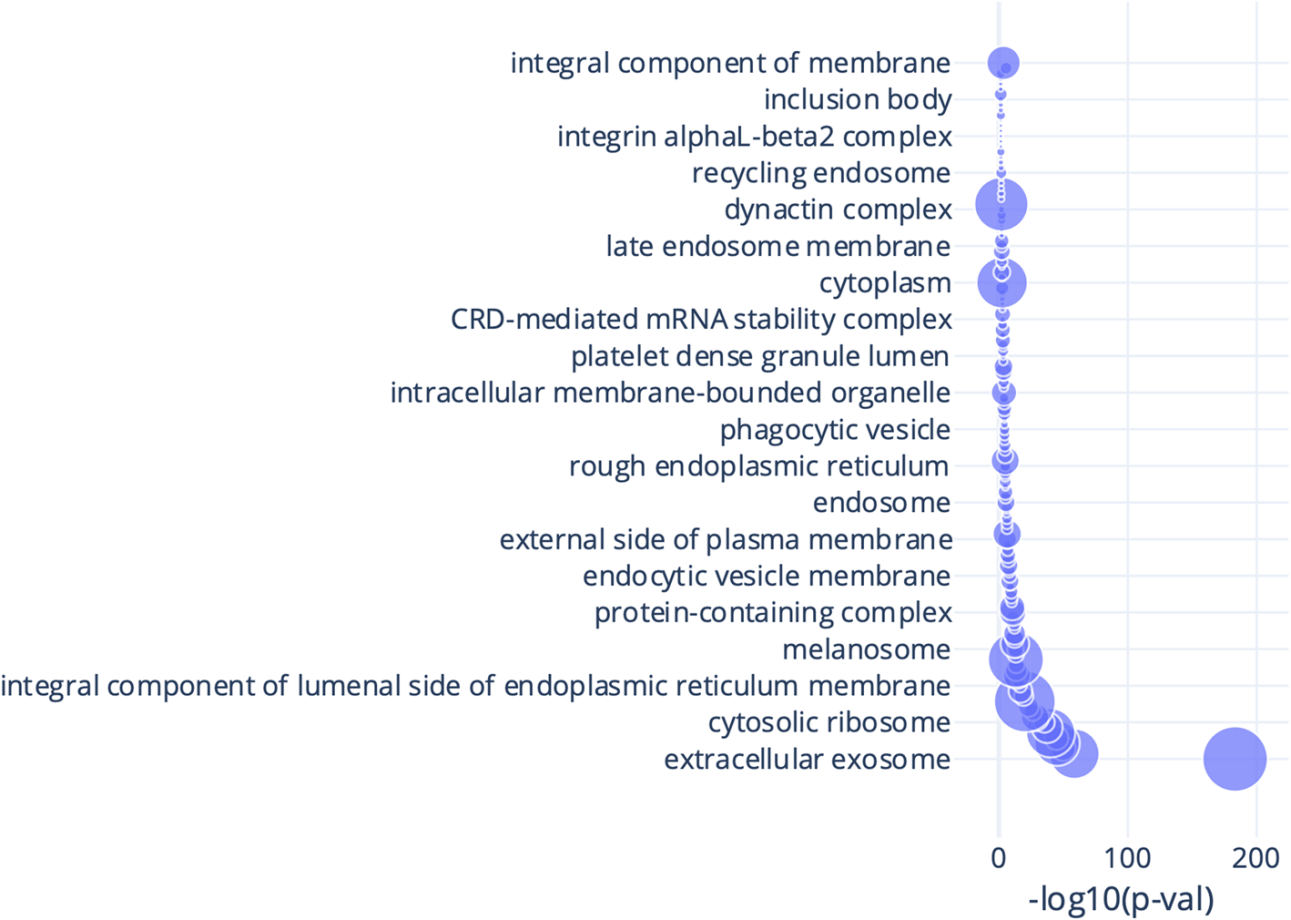
A bubble plot of gene ontology enrichment analysis (GOEA) for the HLA-DR immunopeptidome of total *PBMC* inferred proteins focusing on the cellular component. The x-axis shows a logarithmic transformation of the FDR corrected p-value, while the size of the bubble reflects the number of proteins contributing to each term. Data was generated from 1 × 10^8^ cells and using the protocol described at (Data Generation)

To get a better understanding of the origin of the identified immunopeptidome we used IPTK to integrate the identified immunopeptidomes with sub-cellular compartment data (*Integrating immunopeptidomics and sub-cellular compartment data)* focusing on the replicate with the highest number of unique peptides. As seen in Fig. 5, the majority of proteins have an unknown subcellular location, arguing for the need to better characterize protein subcellular compartment and localization. Interestingly, we observed proteins to be presented and sampled from different cellular compartments, again showing the importance of HLA-II proteins in presenting the protein status of the cell. To understand the contribution of different cellular components to the immunopeptidome we ran a GOEA on the list of inferred proteins (Fig. 6). As seen in the figure, compartment related to extracellular exomes, protein secretions and recycling are highly enriched, which is in agreement with previous findings [44] and with the biological rule of HLA-II proteins as presenters of endosomal and lysosomal proteins.

**Fig. 7:**
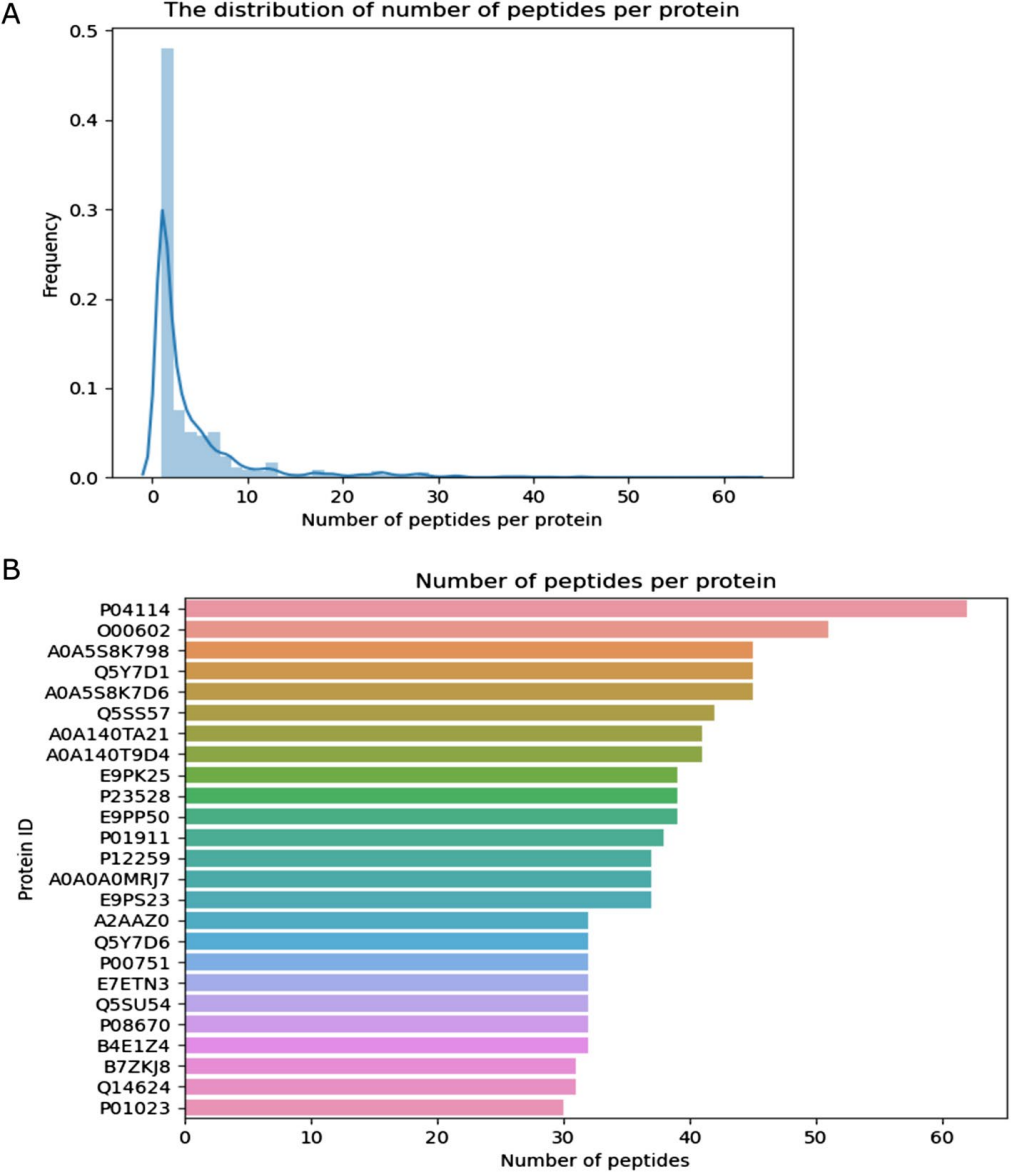
The distribution of number of immunopeptides per protein inferred from the HLA-DR immunopeptidome of total PBMC using 1 × 10^8^ cells as a starting material. **A** Is a density plot showing the distribution of the number of peptides per protein among all inferred proteins. **B** Showing the number of peptides observed in the topmost presented 25 proteins

**Fig. 8:**
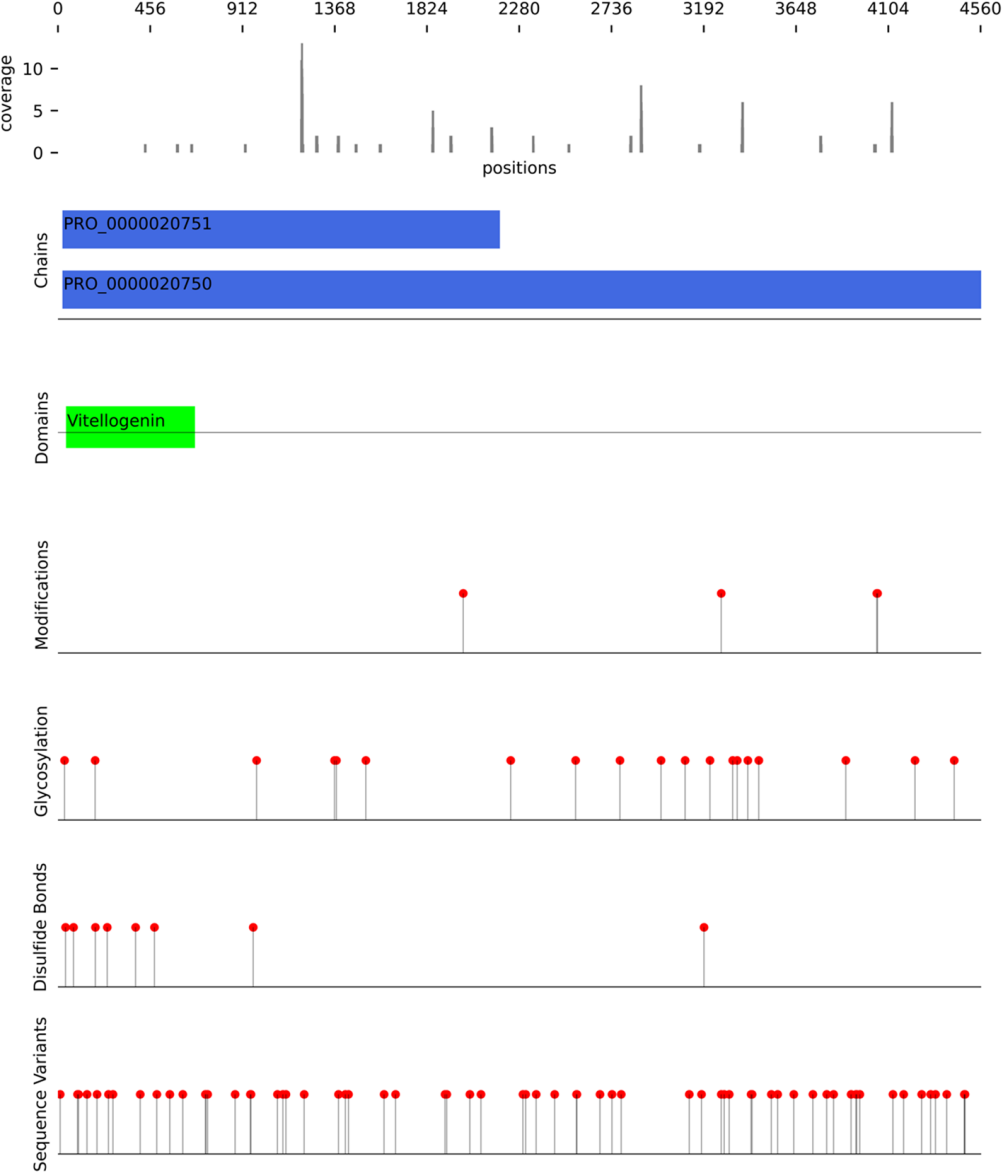
A coverage and annotation plot for Apolipoprotein B-100, UniProt ID: P04114. The coverage track shows the number of peptides obtained from the HLA-DR immunopeptidome of total PBMC using 1 × 10^8^ cells as a starting material. Protein information was obtained from Uniprot database on 5th of December 2020. The chain track shows the location of polypeptide chains in the protein. The “Domain track” shows the position of known domains in the protein backbone. The “Sequence variants track” shows the position of known variants in the protein. The “Glycosylation track” shows the position of the known glycosylation in the protein, while the “Modifications track” shows the position of any known post-translational modification (PTM) in the protein

Next, we used IPTK to study the distribution of the number of peptides per inferred protein (Fig. 7). As shown in Fig. 7A, the majority of proteins have support from only one peptide. However, some proteins have support from a large number of peptides (Fig. 7B). In order to get a deeper understanding of this subset of highly presented proteins, we used IPTK interface to UniProt to leverage preexisting knowledge with the observed coverage, focusing on the protein with the highest number of peptide support, P04114. A *coverage-and-annotation* plot for the protein is shown in Fig. 8. As shown in the figure, the protein is highly glycosylated and has a large number of disulfide bonds which might influence its processing and presentation by the HLA-II machinery, adding a next layer of complexity and control in shaping HLA-II immunopeptidomes. Interestingly, the protein appears also to exhibits a high degree of variations. Implying that a more personalized sequence database, for example, following a proteogenomic approach, is highly desirable to improve immunopeptide identification by capturing peptides that would be missed by using reference databases.

**Fig. 9:**
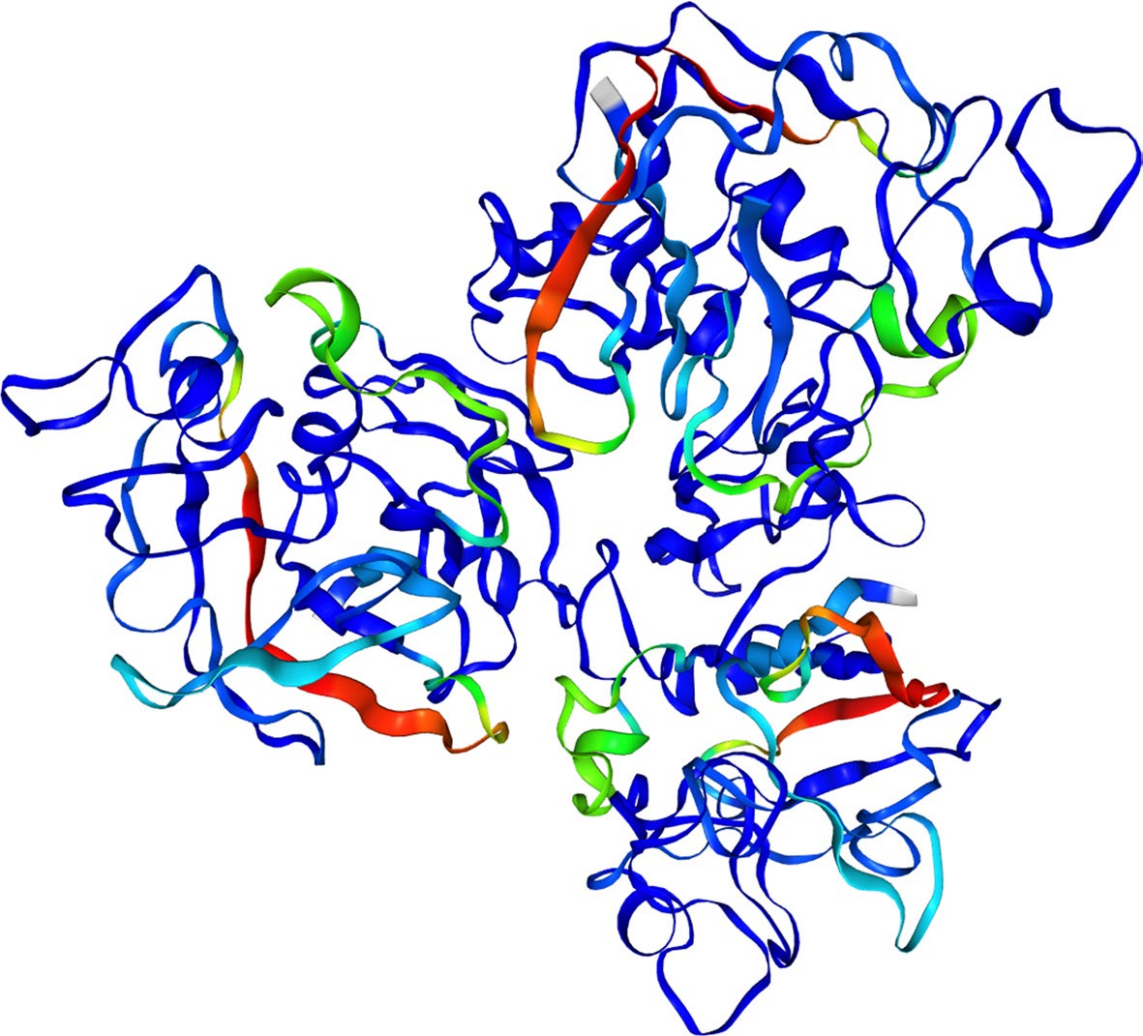
An imposed representation of Ficolin-1 (UniProt ID: O00602 with the corresponding PDB id: 2D39). The color gradients represent the coverage at each position where blue represent low (coverage = 0) while red represent the highest coverage (coverage = 27)

Finally, to understand where the observed peptides are located in the 3D structure of the protein, we used IPTK interface to the protein databank (*Integrating immunopeptidomics and protein structure)* along with the coverage *arra*y of the protein to produce an imposed representation. However, given that the structure of Apolipoprotein B-100 (PO4114) is not currently available, we focused on the second most covered protein Ficolin-1 (O00602) (Fig. 9). As shown in the figure, peptide presentation appears to stem from specific regions (shown in red) on the protein and gradually decrease (shown in green) around this presentation spot until it becomes undetectable, i.e., not presented (shown in blue). A plethora of factors can control this behaviour, for example, the processing machinery, post-translational modification, competition with other peptides and the affinity toward the HLA proteins.

### Use case 3: developing an interactive dashboard

As explained above, IPTK is a toolbox that can be used to analyze immunopeptidomes using Python scripting, or it can be employed for developing other tools and functions. To demonstrate this, we used *Dash* framework from *Plotly* [28] to build a dashboard that can be used to analyze and inspect immunopeptidomics data without any scripting. The graphical user interface (GUI) consists of four main panels. First, the input panel which asks the user to upload a table containing the identified peptides in a user defined format, the sequence database which is a FASTA file containing the source protein sequence, the tissue name and, optionally, HLA-alleles, a gene expression table and a protein localization table (Additional file 1: Fig. S9). The program uses these data to generate an instance of class *Experiment* which is the working engine for the rest of the panels.

The second panel is the visualization panel, which can be used to visualize different aspects of the provided data, for example, the number of peptides per-protein, the number of peptides per subcellular location, et cetera (Additional file 1: Fig. S10A). The third panel is the filter panel which can be used to remove peptides belonging to one or more of the organisms inferred from the provided data. Finally, the coverage panel which can be used to visualize the peptide-coverage of the inferred proteins (Additional file 1: Fig. S10B).

## Discussion

As shown here with different use-cases, IPTK library provides a powerful and extendable framework for combining the output of immunopeptidomic identification pipelines with different omics layers for a rich and in-depth analysis of the identified peptides. The library introduces a wide array of utility functions that can be used to analyze the data at the peptide, the protein, and the experiment level along with classes and methods to compare and integrate the results of different experiments. Due to the modular nature of the library, further extension can be built on top of it to extend and enhance its functionality.

Currently, a potential limitation of the library is the scalability, which might impact the performance, especially with regard to integrating and comparing multiple experiments, i.e., when hundreds of experiments are analyzed simultaneously. Currently, two methods are used to enhance IPTK performance, first, just-in-time compilation using *Numba* [46], which is mainly used to enhance numerical computations. Second, multiprocessing which is used to distribute the work, i.e. the computational load, among multiple CPU cores enabling multiple datasets to be processed on parallel. Nevertheless, current versions of *Numba* offer support for a subset of python constructs, while multiprocessing can be memory-inefficient and computationally heavy. Thus, future releases of the library will aim to improve the performance by reimplementing the computationally intensive tasks in Rust language and bind it to the library. Nevertheless, under the current scale of experiments, i.e., with tens of experiments, IPTK operates seamlessly on a regular desktop computer.

## Conclusion

In conclusion, we believe that the library is a valuable tool for studying and comparing immunopeptidomes and for enriching the analysis by integrating different omics layers using a flexible and modular design that accommodate future extensions. Beside working to improve speed and efficiency, future work should focus on improving data integration. This can be achieved within the IPTK framework by implementing interface to integrate other omics data, for example, genomics, proteomics and metabolomics. Thus, enabling a much deeper understanding of HLA peptide presentation and immunopeptidomes formation. Finally, one important future direction will be adding support for running protein inference on the identified immunopeptidomes, along with support for quantitative immunopeptidomics.

## Supplementary Information

**Additional file 1.** Supplementary Materials and Figures.

## Data Availability

All the source code of the library is available at https://github.com/ikmb/iptoolkit. The mass spectrometry proteomics data have been deposited to the ProteomeXchange [47] Consortium via the PRIDE [48] partner repository with the dataset identifier PXD023032 and https://doi.org/10.6019/PXD023032.

## Abbreviations

ACN: Acetonitrile
DNA: Deoxyribonucleic acid
GOEA: Gene ontology enrichment analysis
GUI: Graphical user interface
HLA: Human leukocyte antigen
IPTK: Immunopeptidomics toolkit library
LC–MS/MS: Liquid chromatography with tandem mass spectrometry
IO: Input/output
ID: Identity
LRS: Leukocyte reduction system
MEME: Multiple expectation maximizations for motif elicitation
MS: Mass spectrometry
MDS: Multidimensional scaling
SLE: Systemic lupus erythematosus
TFA: Trifluoroacetic acid
PTM: Post-translational modification
PDB: Protein data bank
PBMC: Peripheral blood mononuclear cells
PyPI: Python package index
RNA: Ribonucleic acid
