# Supplementary material for: Immunopeptidomics toolkit library (IPTK): a python-based modular toolbox for analyzing immunopeptidomics data
Source: BMC Bioinformatics. 2021 Aug 17;22:405. doi: 10.1186/s12859-021-04315-0 (PMC8369717; doi:10.1186/s12859-021-04315-0)
Supplement: Supplementary file 1 — Additional file 1. Supplementary Materials and Figures. [file 12859_2021_4315_MOESM1_ESM.docx]

**Supplementary Materials**

***Data Generation***

During the development and testing of the library two datasets were used. The first is the HLA-Ligand Atlas database Release 2020.06 [44]. The second, is an internal dataset containing the HLA-DR immunopeptidome of total PBMC of one donor using two biological replicates.

For each replicate $5x{10}^{7}$and $1x{10}^{8}$cells were lysed, and HLA-DR was pulled down using L243 antibody (Bio X cell). Peptides were released from HLA-DR using 0.1 M acetic acid and purified using C18 purifications columns.

Dried peptide samples were resuspended in 3% acetonitrile (ACN) and 0.1% trifluoroacetic acid (TFA) and injected on a Dionex Ultimate 3000 nano-UHPLC coupled to a Q Exactive Plus mass spectrometer (Thermo Scientific, Bremen). The samples were washed on a trap column (Acclaim Pepmap 100 C-18, 5 mm × 300 μm, 5 μm, 100 Å, Dionex) for 2 minutes with 2% ACN/0.05% TFA at a flow rate of 20 μl/minute prior to peptide separation using an Acclaim PepMap 100 C-18 analytical column (50 cm × 75 μm, 2 μm, 100 Å, Dionex). Peptides were separated over a 90-minutes gradient (5-30%B) at a flow rate of 300 nL/min using eluent A (0.05% formic acid) and eluent B (80% ACN/0.04% formic acid). Full scan MS spectra were acquired between 250 and 1,800 m/z at a resolution of 70,000 at m/z 200. The ten most intense precursors with charge states greater than 2+ were selected with an isolation window of 2.1 m/z and fragmented by HCD with normalized collision energies (NCE) of 27.5 at a resolution of 17,500. Maximum MS2 ion injection time was set to 200 milliseconds. Lock mass (445.120025) and dynamic exclusion (20 seconds) were enabled.

Peptide identification was carried out using *MHCQuant* [24] using the human reference proteome available on UniProt [36]. With a peptide-level false discovery rate of 5% and peptide length range of 9 to 27 amino acids, all other values were set to their default values.

***Supplementary Figures***


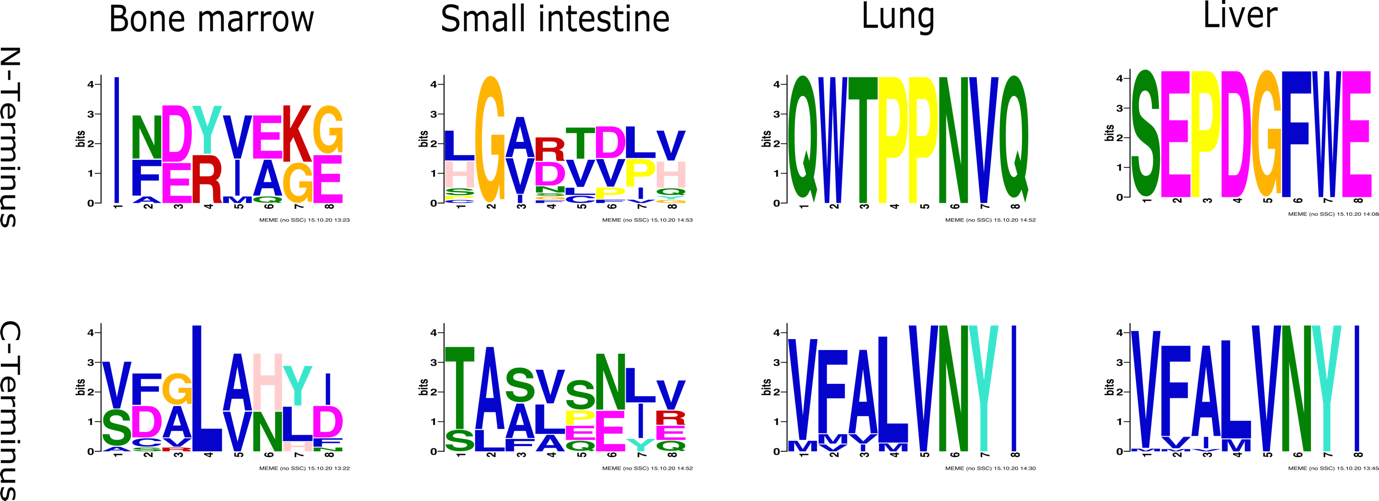


**Fig. S1:** Examples of a motif-discovery analysis using 10-mers located upstream and downstream of the HLA-II peptidome of four different tissues from donor AUT01-DN06. The peptidome data was obtained from HLA-ligand atlas (Release 2020.06). The motifs were computed using MEME 5.1 using the default settings. The number of peptides and proteins per tissue HLA-II peptidome were as follow; Bone marrow: 292 peptides belonging to 152 proteins, small intestine: 213 peptides belonging to 108 proteins, lung: 5845 peptides belonging to 1305 proteins and finally liver: 3913 peptides belonging to 970 proteins.


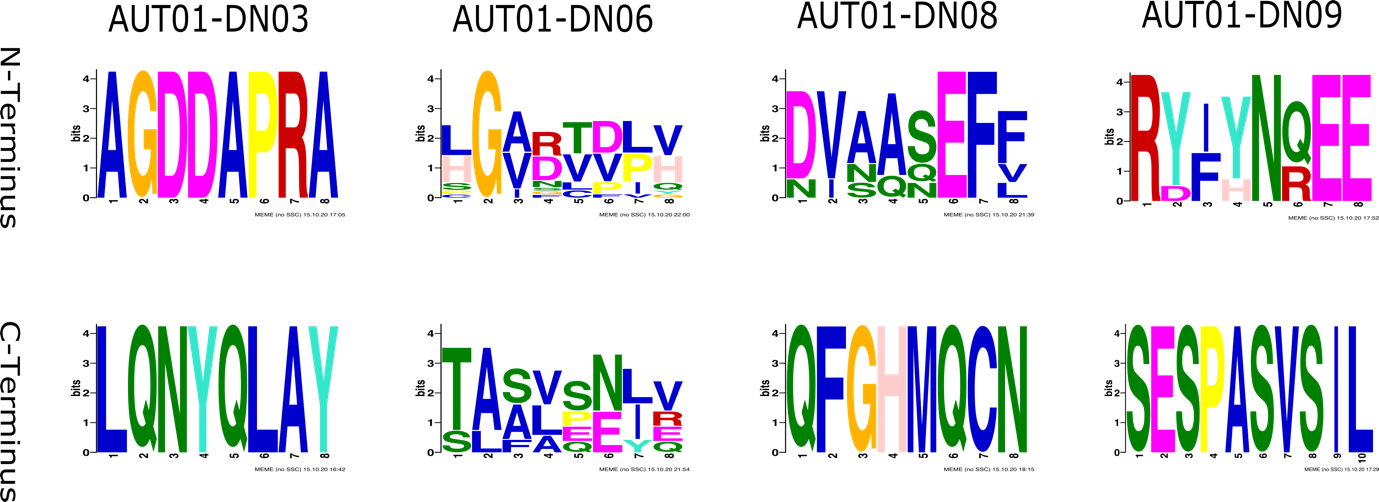


**Fig. S2:** Example of a motif-discovery analysis of the 10-mers located upstream and downstream of the HLA-II peptidome of the small intestine of different donors. The number of peptides and proteins for each proband were as follow; AUT01-DN03: 3167 peptides belonging to 810 proteins, AUT01-DN06: 213 peptides identified from 108 proteins, AUT01-DN08: 4684 peptides belonging to 1080 proteins, AUT01-DN09: 1569 peptides belonging to 452 proteins. The peptidome data was obtained from HLA-ligand atlas (Release 2020.06). The motifs were computed using MEME 5.1 using the default settings.


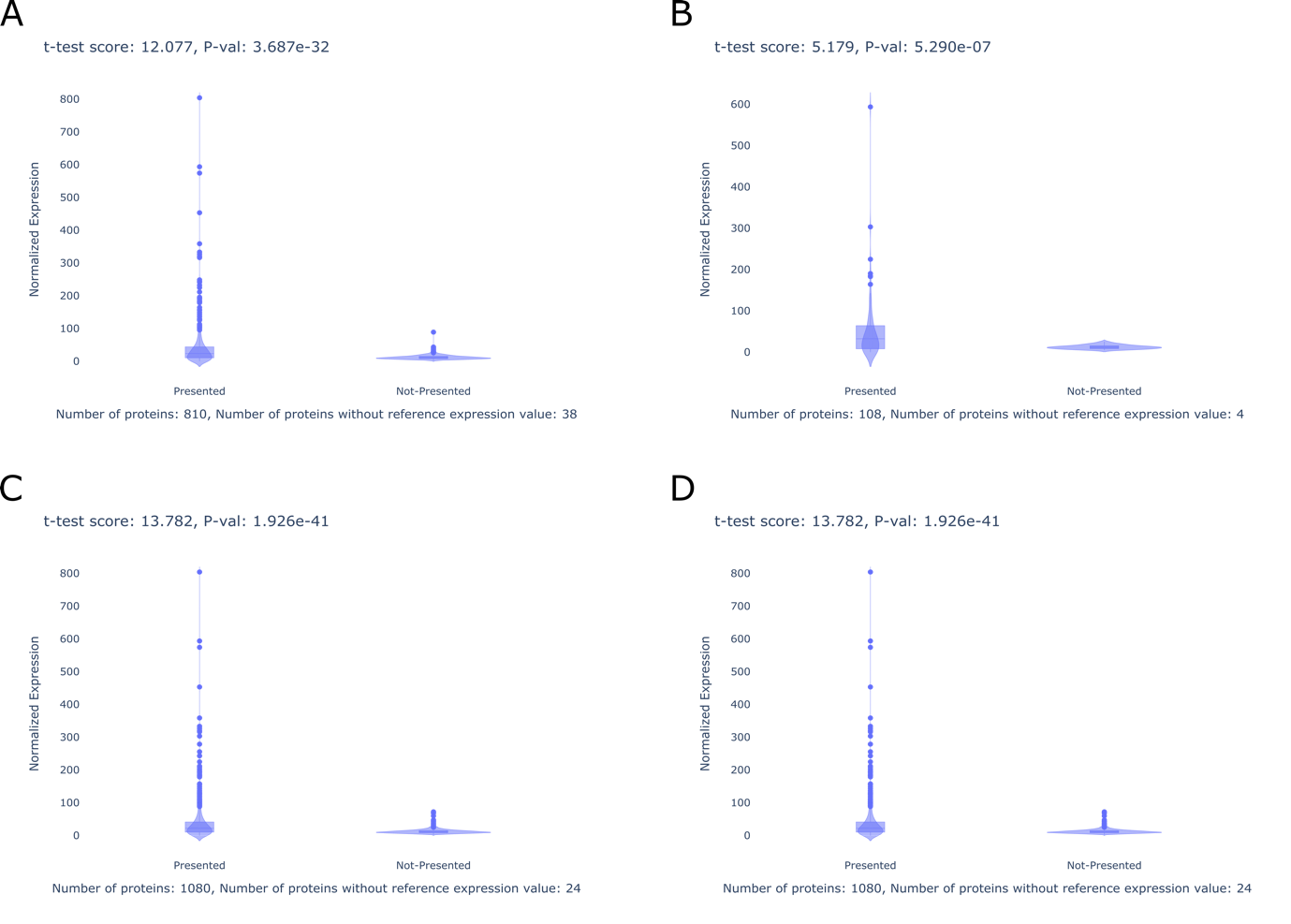


**Fig. S3:** Comparison between the gene expression of proteins contributing to the HLA-II peptidome, referred to as ‘Presented’ in the figure and non-contributing proteins, referred to as ‘Not-Presented’. Gene expression was computed using the method: ´get_expression_of_parent_proteins` which is a part of the Experiment API and visualization of each figure was performed using the function: ´plotly_parent_protein_expression_in_tissue` which is part of the visualization module. Gene expression data was accessed from the Human protein Atlas [41]. (A) shows the results for the small intestine HLA-II peptidome of proband AUT01-DN03, (B) for AUT01-DN06, (C) for AUT01-DN08 and (D) for AUT01-DN09. The peptidome data was obtained from HLA-ligand atlas (Release 2020.06).


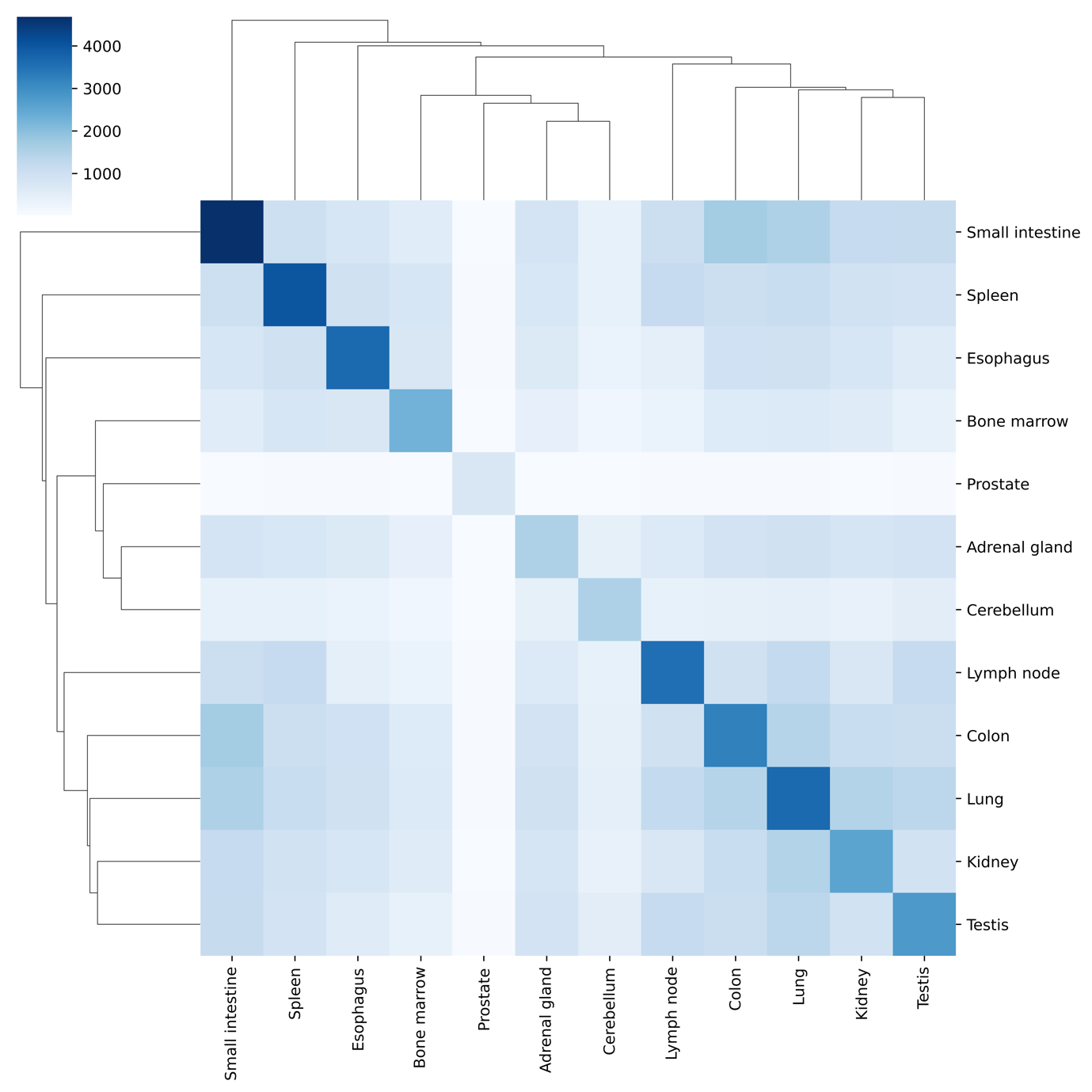


**Fig. S4**: Peptide overlap among HLA-II peptidomes of different tissues obtained from proband AUT01-DN08 where peptide overlap is defined as the presence of the same peptide in the peptidome of two different tissues. The color gradient reflects the number of overlapped peptides. The immunopeptidomes were obtained from HLA-ligand atlas (Release 2020.06).


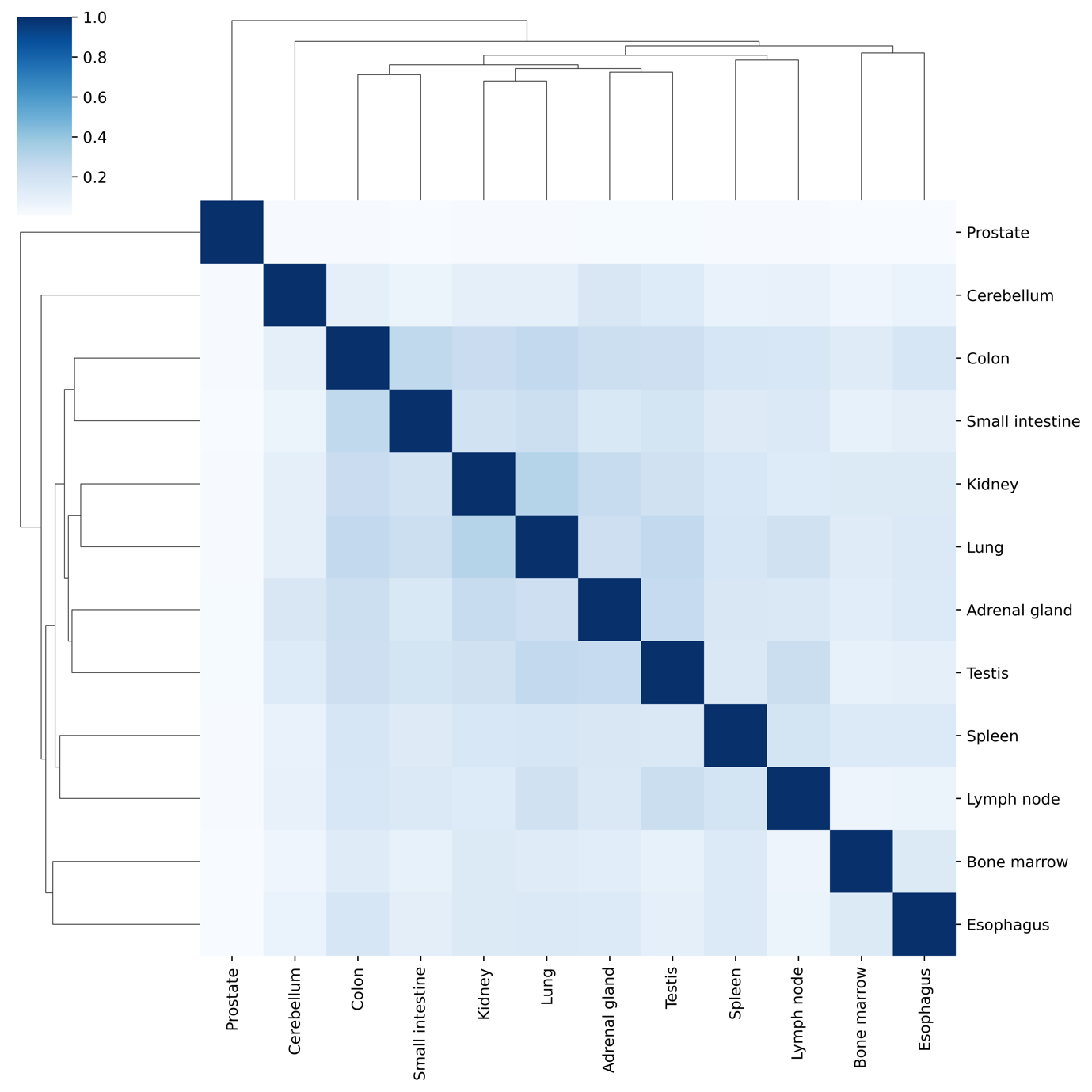


**Fig. S5**: Peptide-level Jaccard index among HLA-II immunopeptidomes of different tissues obtained from proband AUT01-DN08. The color gradient reflects Jaccard-index computed at the level of identified peptides between the immunopeptidome of pairs of tissues. The immunopeptidomes were obtained from HLA-ligand atlas (Release 2020.06).


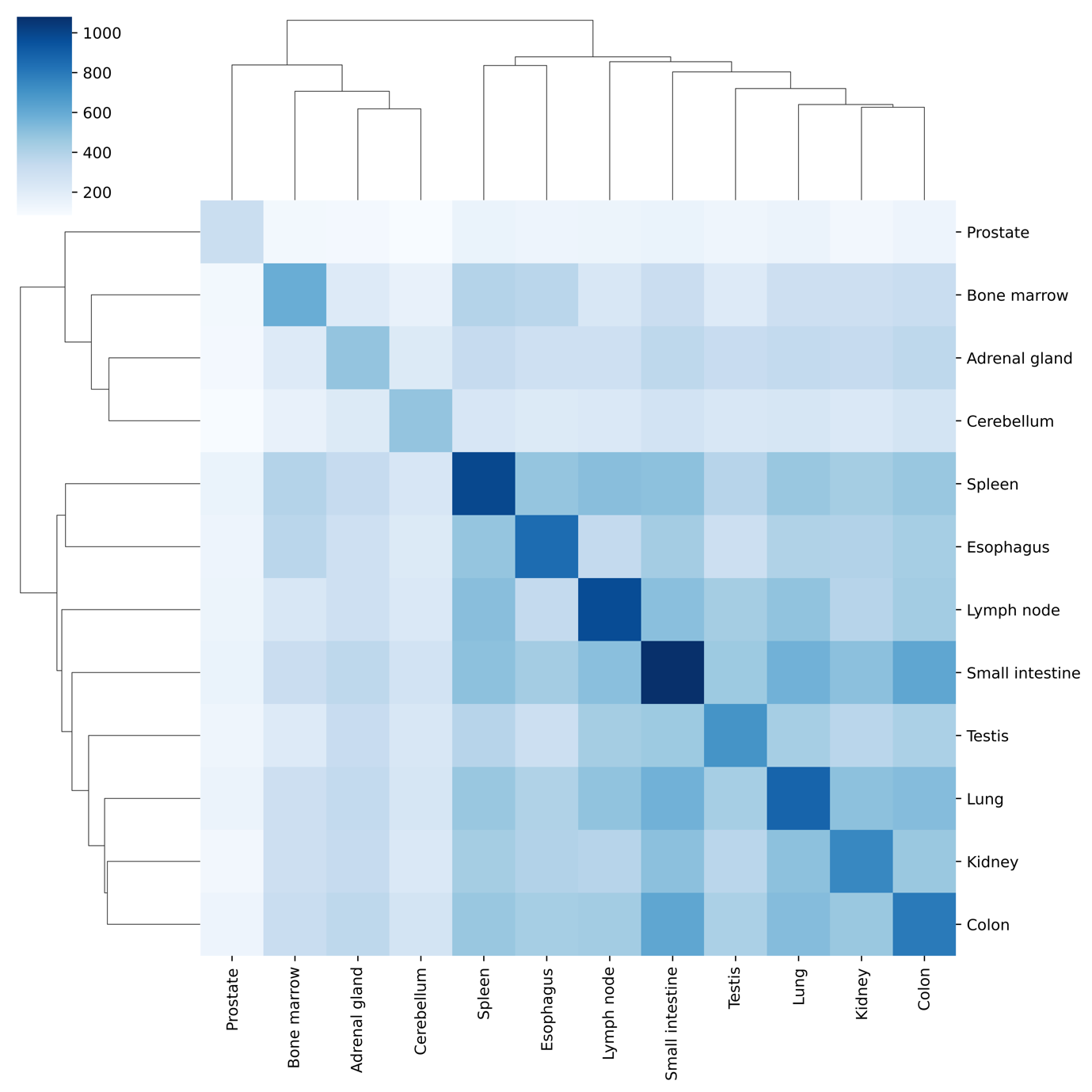


**Fig. S6**: Protein overlap among HLA-II peptidomes of different tissues obtained from proband AUT01-DN08 where protein overlap is defined as the presence of the same protein in the immunopeptidome of two different tissues. The color gradient reflects the number of overlapping proteins. The immunopeptidomes were obtained from HLA-ligand atlas (Release 2020.06).


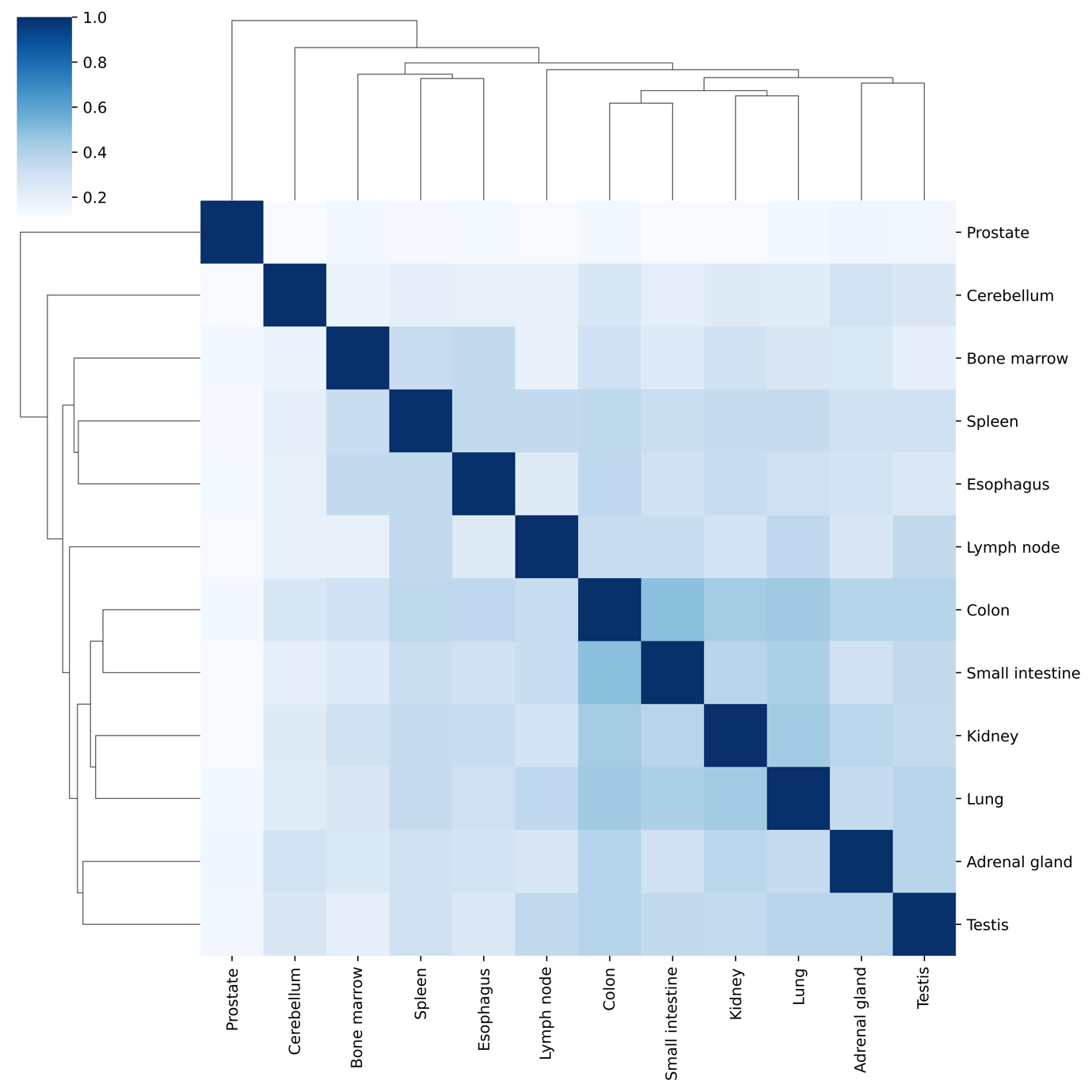


**Fig. S7**: Protein-level Jaccard index among HLA-II peptidomes of different tissues obtained from proband AUT01-DN08. The color gradient reflects Jaccard-index computed at the level of inferred proteins between the immunopeptidome of pairs of tissues. The immunopeptidomes were obtained from HLA-ligand atlas (Release 2020.06).


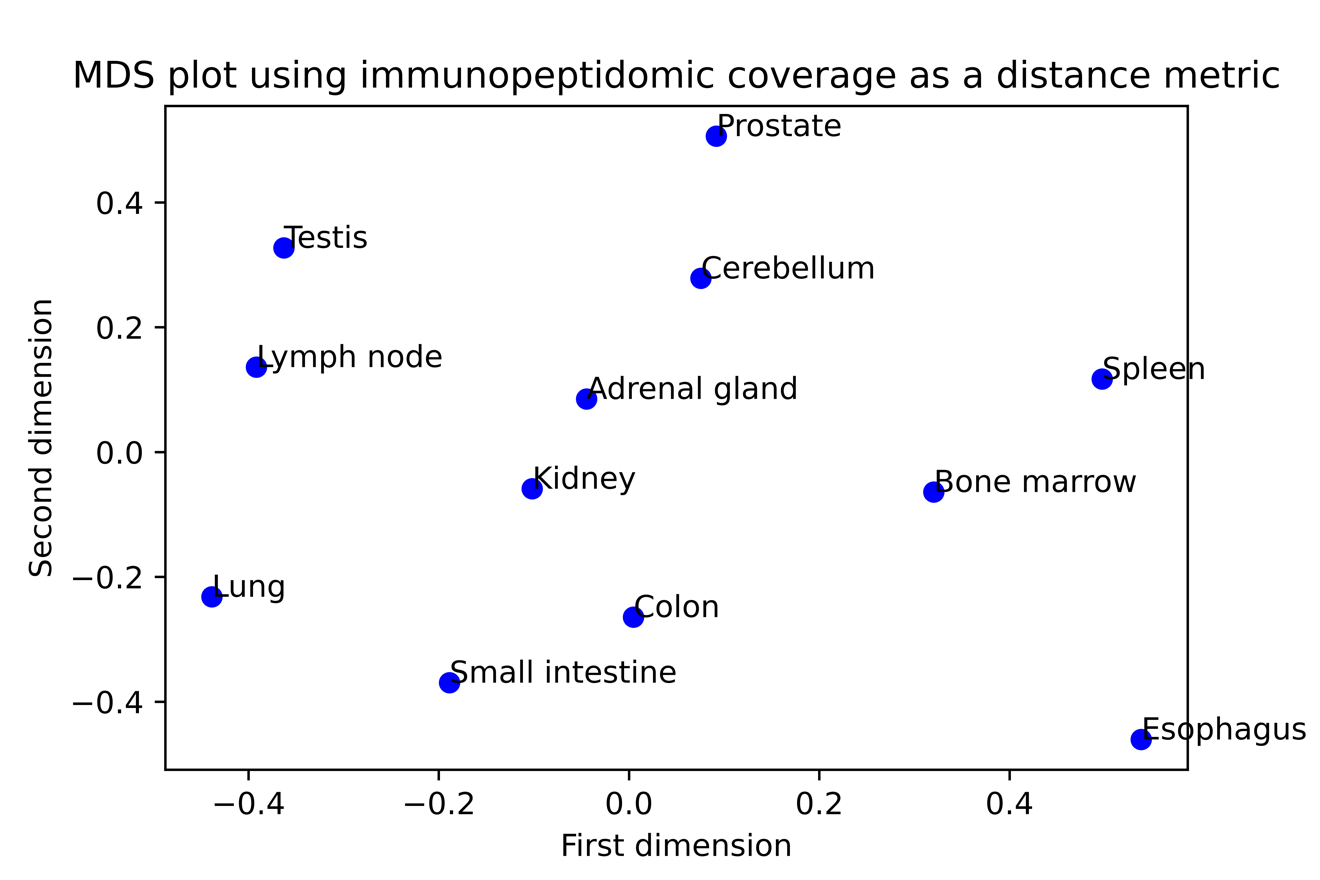


**Fig. S8**: MDS plot for HLA-II peptidomes of different tissues obtained from proband AUT01-DN08 using immunopeptidomics coverage as a distance metric. The immunopeptidomes were obtained from HLA-ligand atlas (Release 2020.06).


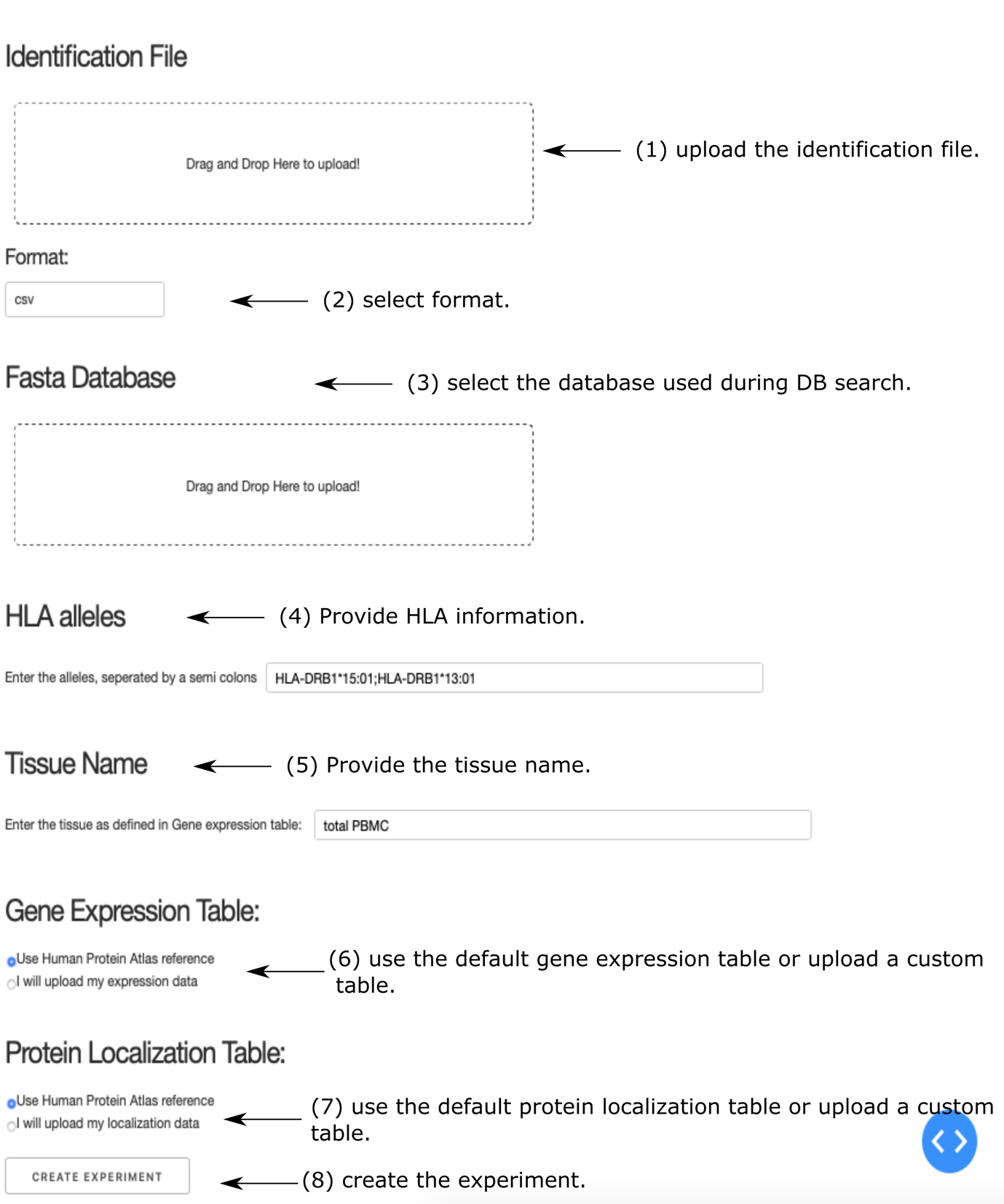


**Fig. S9**: An overview of the Input panel for the GUI Application.

***
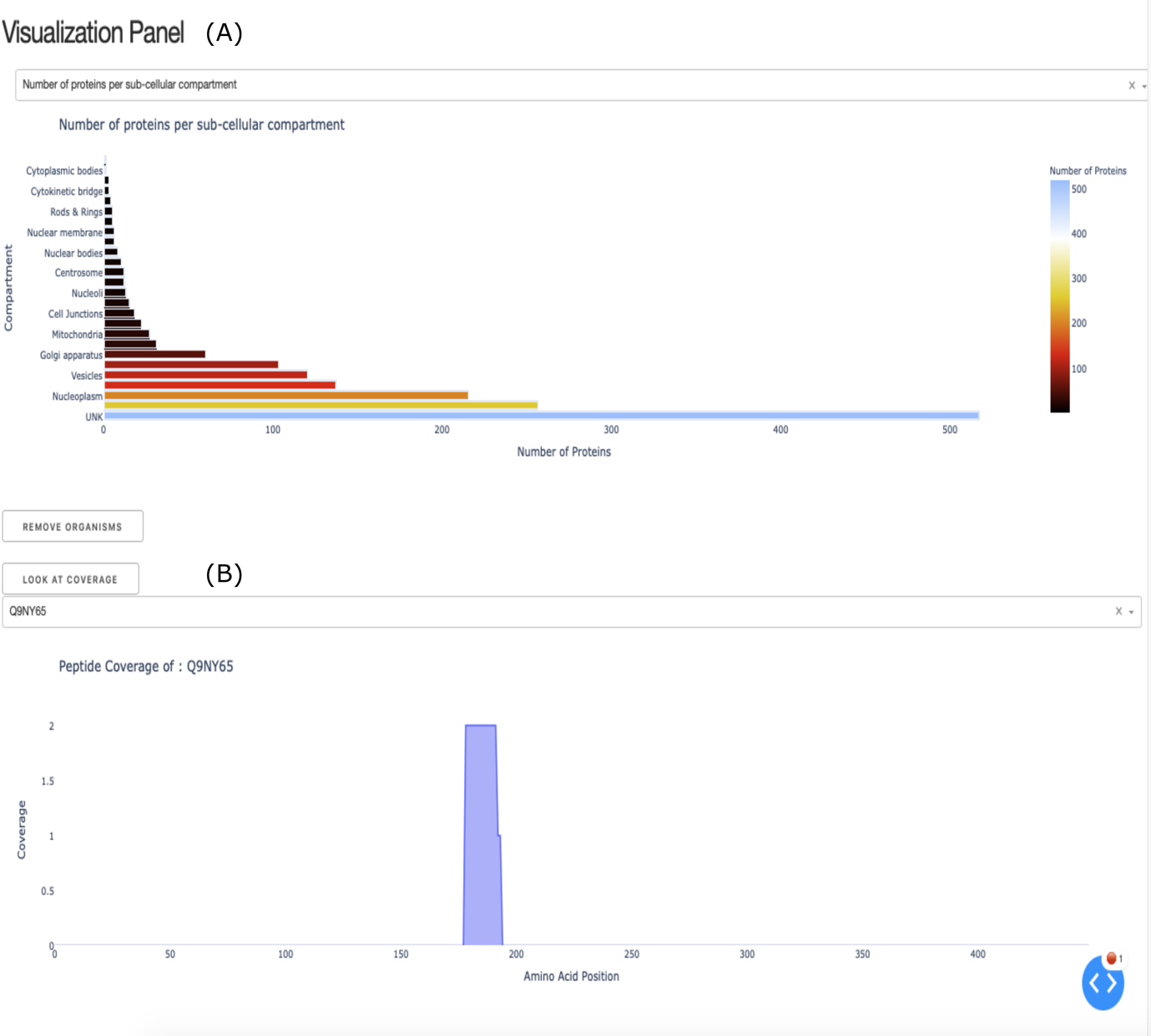
***

**Fig. S10**: An overview of GUI Application. (A) shows the visualization panel, here depicting the number of parent proteins per subcellular compartment. The coverage panel (B) shows the coverage of an exemplarily protein, Q9NY65, by the identified peptides.
